# Usefulness of PET/CT for diagnosis of periosteal chondrosarcoma of the femur: A case report

**DOI:** 10.3892/ol.2014.2010

**Published:** 2014-03-28

**Authors:** SHOTA MORIMOTO, HIROYUKI FUTANI, KONAN TSUCHIYAMA, SATORU FUKUNAGA, YOSHITANE TSUKAMOTO, SHINICHI YOSHIYA

**Affiliations:** 1 Department of Orthopedic Surgery, Hyogo College of Medicine, Nishinomiya, Hyogo 663-8501, Japan; 2 Department of Pathology, Hyogo College of Medicine, Nishinomiya, Hyogo 663-8501, Japan

**Keywords:** chondrosarcoma, PET, imaging, periosteal tumor

## Abstract

Periosteal chondrosarcoma is an extremely rare low-grade malignant cartilaginous tumor arising from the external bone surface. Diagnosis of periosteal chondrosarcomas may be challenging, since this condition closely resembles periosteal chondromas. It has been reported that positron emission tomography (PET) is useful in distinguishing benign from malignant cartilaginous tumors using a maximum standardized uptake value (SUV_max_) cut-off of 2.0 or 2.3. This report presents the case of a 40-year-old female with an 18-month history of a tender mass in the left distal femur. Radiological findings demonstrated periosteal buttressing. Magnetic resonance imaging (MRI) revealed a chondrogenic tumor of 3 cm in size developing from the external bone surface. It was difficult to differentiate periosteal chondrosarcoma from periosteal chondroma on the basis of size and the radiological and MRI findings. PET/computed tomography (CT) revealed abnormal linear uptake with an SUV_max_ of 2.7, indicating a malignant tumor. A diagnosis of periosteal chondrosarcoma was made, and wide resection was performed. Tumor histology was consistent with grade II chondrosarcoma. PET/CT is thus useful in differentiating periosteal chondrosarcoma from periosteal chondroma.

## Introduction

Periosteal chondrosarcoma is an extremely rare and low-grade malignant cartilaginous tumor arising from the external surface of bones (1), and it accounts for <2% of all chondrosarcomas (2). Wide resection is the only recommended treatment, as it minimizes the potential for recurrence and metastases (3–6). Therefore, the pre-operative diagnosis of periosteal chondrosarcoma is important. However, periosteal chondrosarcomas can be difficult to differentiate from benign periosteal chondromas, since these two conditions share features on imaging and histological examination (5).

Fluorine-18 fluorodeoxyglucose (^18^F-FDG) positron emission tomography (PET) is a diagnostic imaging technique that detects glucose uptake by cells with high metabolic activity, including the heart, brain and tumor cells. The usefulness of PET has been reported in diagnosing malignant cartilaginous tumors, particularly those with borderline histological, imaging and clinical characteristics (7–9). Feldman *et al* (7) reported that a maximum standard uptake value (SUV_max_) cut-off of 2.0 could be used to distinguish benign from malignant cartilaginous tumors. However, there have been no reports on the use of PET for diagnosing periosteal chondrosarcoma due to the rarity of this condition.

The present study reports a case of femoral periosteal chondrosarcoma that was diagnosed by PET/computed tomography (CT). Written consent was obtained from the patient and the patient’s family for the publication of this study.

## Case report

A 40-year-old female presented with an 18-month history of a tender mass in the left distal femur. Physical examination revealed a bony hard mass in the medial aspect of the metaphysis in the distal femur.

**Figure 1:**
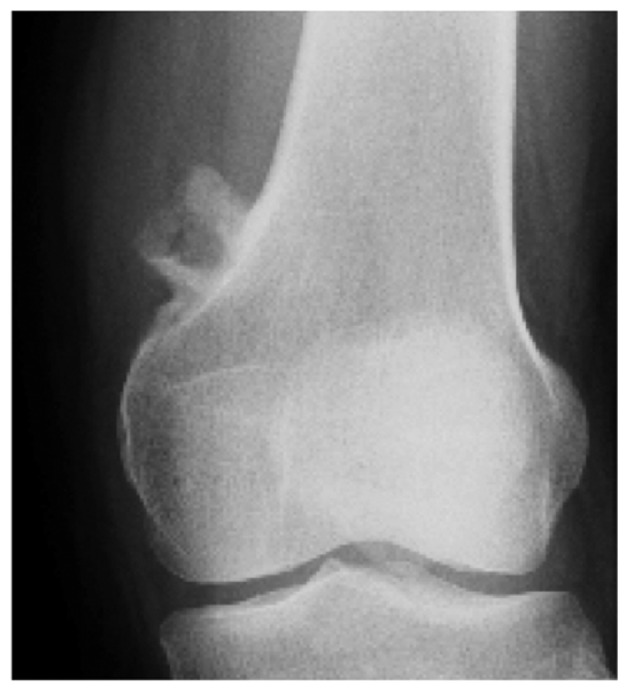
Anteroposterior radiograph revealing a well formed sclerotic periosteal reaction in the metaphysis of the distal femur.

**Figure 2:**
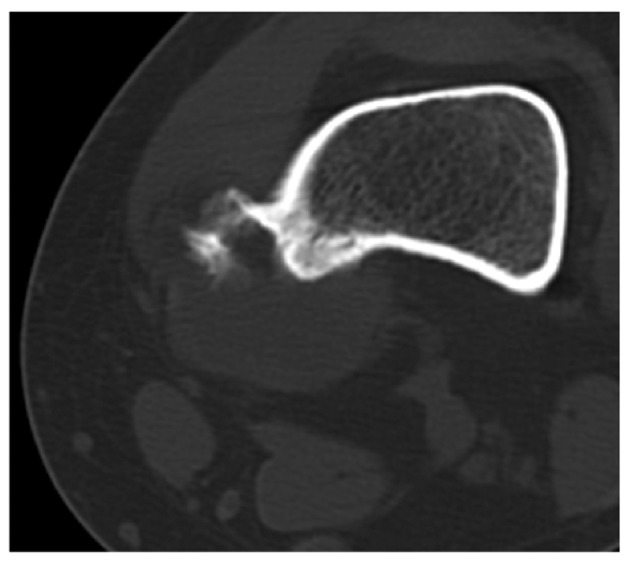
Axial computed tomography revealing that the tumor contained calcific densities characteristic of the external bone surface and associated with a thickened cortex, which indicates a periosteal chondrogenic tumor.

**Figure 3:**
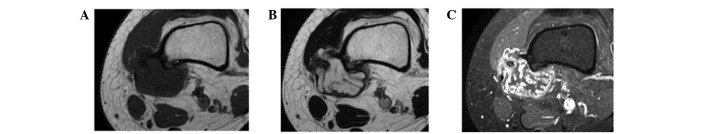
(A) Axial T1-weighted MRI showing a sharply delineated mass 3 cm in diameter with low to intermediate signal intensity in the bone surface of the metaphyseal region. (B) Axial T2-weighted MRI with a bright signal and associated lobulated structure with hypo-intense septa. (C) Gadolinium-enhanced T1-weighted MRI with peripheral and septal enhancements. No intramedullary extension or edema was identified.

**Figure 4:**
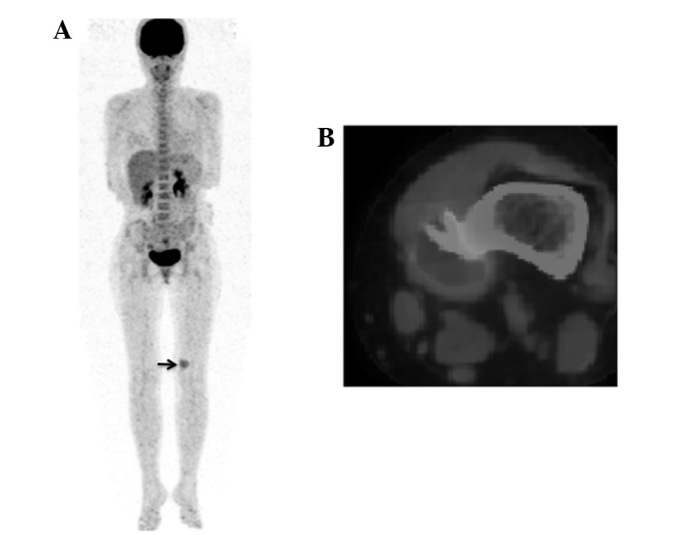
(A) Whole-body PET revealing abnormal ^18^F-fluorodeoxyglucose uptake in the distal aspect of femur (arrow). No evidence of metastasis was identified. (B) PET/computed tomography of the distal femur showing periosteal buttressing with abnormal uptake, indicating a malignant cartilaginous tumor. PET, positron emission tomography.

Radiography revealed a well formed sclerotic periosteal reaction, indicating periosteal buttressing (Fig. 1). CT images clearly revealed a tumor containing calcific densities characteristic of the external bone surface, which was a juxtacortical lesion associated with a thickened cortex. The tumor had no connection with bone marrow and a periosteal tumor was suspected (Fig. 2). Magnetic resonance imaging (MRI) revealed a sharply delineated mass at the bone surface, measuring 3 cm in diameter. An area of low to intermediate signal intensity was present on T1-weighted images. On T2-weighted images, the tumor had a bright signal and an associated lobulated structure with hypo-intense septa. Gadolinium-enhanced T1-weighted images revealed peripheral and septal enhancement. No intramedullary extension or edema was identified. MRI findings indicated a chondrogenic tumor at the external bone surface, which again suggested a periosteal chondroma or a periosteal chondrosarcoma (Fig. 3). PET images revealed abnormal ^18^F-FDG uptake in the distal aspect of the femur. Furthermore, PET/CT images clearly demonstrated periosteal buttressing with an abnormal ^18^F-FDG SUV_max_ of 2.7, indicating a malignant tumor of the bone surface in the distal femur. In addition, no evidence of distant metastases was identified (Fig. 4).

**Figure 5:**
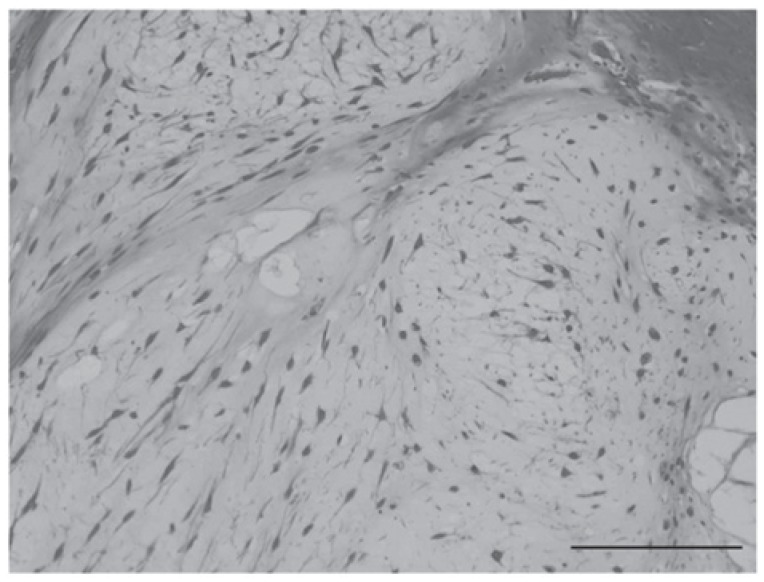
Histological examination revealing atypical spindle cells proliferating in a myxomatous background. Cellular atypia and marked myxomatous change indicated grade II chondrosarcoma (bar, 200 μm). Magnification, ×100.

En bloc resection of the tumor was performed with a wide margin. The histology of the resected sample demonstrated variant forms of nuclei, the appearance of cells with two nuclei, and cytostromatic changes such as myxoma. Periosteal chondrosarcoma grade II was diagnosed (Fig. 5).

The patient started walking on the day following surgery. Neither recurrence nor metastases have been identified in the 3 years following surgery.

## Discussion

Periosteal chondrosarcoma presents in the second to fourth decade of life as a slowly growing painless mass. The most common site of this tumor is the distal femur, followed by the proximal humerus. The size is generally >5 cm (3). Radiographic features are cortical saucerization, cortical thickening, cortical marginal buttressing and a soft tissue mass that may contain matrix calcification (10). Invasion of the medullary cavity is infrequent, but has been previously described (5,11). In the treatment of this condition, wide resection is essential since the rates of recurrence and metastases are higher for patients treated with intralesional or marginal excisions than for patients treated with wide resection. Papagelopoulos *et al* (2) reported that the 5-year local recurrence-free survival rate was lower in patients treated with intralesional or marginal excisions (25%) than for patients treated with wide resections (93%). In addition, the 5-year metastasis-free survival rate was lower for patients who underwent intralesional or marginal excisions (50%) than for patients who were treated by wide resection (100%).

Periosteal chondroma is the benign counterpart of periosteal chondrosarcoma. Periosteal chondroma is generally a smaller painless mass <3 cm in size. Local excision is sufficient treatment for chondromas (5). The preoperative differentiation of the conditions is thus important, since the treatments differ.

Radiographically, periosteal chondrosarcoma and periosteal chondroma appear as a saucer-shaped defect with thickening and sclerosis of the underlying cortex. Periosteal chondrosarcoma may invade the underlying cortex and medullary cavity, while medullary invasion is not observed with periosteal chondromas (12–14). Robinson *et al* (5) reported that size is the most reliable indicator for distinguishing the two conditions. Periosteal chondromas are typically small (1–6.5 cm; median, 2.5 cm), whereas periosteal chondrosarcomas tend to be larger (3–14 cm; median, 4 cm). In the present case, the lesion measured ~3 cm in size with no invasion into the underlying cortex and medullary cavity. It was challenging to differentiate periosteal chondrosarcoma from periosteal chondroma on the basis of size and the radiological and MRI findings alone.

Previous studies have reported that PET is a useful imaging method for differentiating between benign and malignant cartilaginous tumors (7–9). An SUV_max_ cut-off of 2.0 can be used to distinguish benign from malignant cartilaginous tumors with 90.9% overall sensitivity, 100% specificity and 96.6% accuracy (7). Lee *et al* (9) reported that grade I chondrosarcoma is difficult to differentiate from chondroma. However, an SUV_max_ cut-off of 2.3 was useful in differentiating grade II or III chondrosarcomas from chondroma and grade I chondrosarcomas. The positive predictive value was 0.82 (95% confidence interval, 0.48–0.97), and the negative predictive value was 0.96 (95% confidence interval, 0.77–1.00).

In the present case, the typical findings of periosteal chondrogenic tumor were identified by radiography, CT and MRI. However, none of these findings could clearly differentiate periosteal chondrosarcoma from periosteal chondroma. However, given the SUV_max_ of 2.7, PET/CT was able to indicate malignancy. Consequently, a periosteal chondrosarcoma of grade II or III was diagnosed and wide resection was therefore carried out. In conclusion, PET/CT can distinguish periosteal chondrosarcoma from periosteal chondroma, even where differentiation of the conditions is challenging on the basis of size and radiographical findings.
